# Low incidence of helminth infections (schistosomiasis, strongyloidiasis, filariasis, toxocariasis) among Dutch long-term travelers: A prospective study, 2008-2011

**DOI:** 10.1371/journal.pone.0197770

**Published:** 2018-05-30

**Authors:** Femke W. Overbosch, Tom van Gool, Amy Matser, Gerard J. B. Sonder

**Affiliations:** 1 Department of Infectious Diseases, Public Health Service (GGD), Amsterdam, the Netherlands; 2 National Coordination Centre for Traveller’s health Advice (LCR), Amsterdam, the Netherlands; 3 Department of Internal Medicine, Division of Infectious Diseases, Tropical Medicine and AIDS, Academic Medical Center, Amsterdam, the Netherlands; 4 Department of Medical Microbiology, Parasitology Section, Academic Medical Center, Amsterdam, the Netherlands; 5 Department of Infectious Disease Research and Prevention, Public Health Service (GGD), Amsterdam, the Netherlands; Uniformed Services University of the Health Sciences, UNITED STATES

## Abstract

**Background:**

Despite the considerable burden of helminth infections in developing countries and increasing international travel, little is known about the risks of infection for travelers.

**Objective:**

We studied the attack and incidence rate of serology confirmed strongyloidiasis, filariasis, and toxocariasis among long-term travelers and associated factors. A second objective was to evaluate eosinophilia as a positive/negative predictive value (PPV/NPV) for a recent helminth infection.

**Methods:**

From 2008 to 2011, clients of the Public Health Service travel clinic planning travel to (sub)tropical countries for 12–52 weeks were invited to participate in a prospective study. Participants kept a weekly diary, recording itinerary, symptoms, and physician visits during travel and completed a post-travel questionnaire. Pre- and post-travel blood samples were serologically tested for the presence of IgG antibodies against *Schistosoma* species, *Strongyloides stercoralis*, filarial species, and *Toxacara* species and were used for a blood cell count. Factors associated with recent infection were analyzed using Poisson regression. Differences among groups of travelers were studied using chi square tests.

**Results:**

For the 604 participants, median age was 25 years (interquartile range [IQR]: 23–29), 36% were male, median travel duration was 20 weeks (IQR: 15–25), and travel purpose was predominantly tourism (62%). Destinations were Asia (45%), Africa (18%), and the Americas (37%).

Evidence of previous infection was found in 13/604 participants: antibodies against *Schistosoma* spp. in 5 (0.8%), against *S*.*stercoralis* in 3 (0.5%), against filarial species in 4 (0.7%), and against *Toxocara* spp. in 1 (0.2%). Ten recent infections were found in 9 participants (3, 1, 6, 0 cases, in the above order), making the attack rates 0.61, 0.17, 1.1 and 0, and the incidence rates per 1000 person-months 1.5, 0.34, 2.6 and 0. The overall PPV and NPV of eosinophila for recent infection were 0 and 98%, respectively.

**Conclusions:**

The risk of the helminth infections under study in this cohort of long-term travelers was low. Routine screening for eosinophilia appeared not to be of diagnostic value.

## Introduction

Being among the most widespread infectious agents in human populations, helminths (i.e., roundworm and flatworm parasites) are an enormous burden for many low-income countries [1, 2]. Millions of people in developing countries are chronically infected with at least one helminth species [1]. Infection can produce a wide range of illnesses, depending on the involved species. The World Health Organization was requested by its World Health Assemby in 1974 to intensify research into the major tropical parasitic diseases [3]. Since then, several programs regarding helminths have been launched, like the Onchocerciasis Elimination Program for the Americas (OEPA, 1993), African Programme for Onchocerciasis Control (APOC, 1995), Global Programme to Eliminate Lymphatic Filariasis (GPELF, 2000), and Schistosomiasis Control Initiative (SCI, 2002) [4–8]. Several such programs include mass drug administration (MDA) which often can prevent and alleviate symptoms of disease and reduce infection prevalence to levels that mitigate transmission and new infections [9]. MDA proved to be an effective global public health control measure that could by-pass the cost of screening diagnostics and use drugs donated by pharmaceutical companies [1, 10]. However, while important progress was made, the global burden of schistosomiasis, for example, is still estimated at 3.5 million disease-adjusted life-years (DALYs) and for lymphatic filariasis, it is more than 2 million DALYs [2]. Although complete elimination of helminth infections will depend amongst others on mosquito-control, improvement of sanitation, and access to clean water, fundamental research is still needed to develop alternative treatment or medication targeting various stages of the parasites [1, 8, 9].

Travelers to helminth-endemic countries may be at risk for contracting helminth infections, for example, when they are exposed to vectors and/or engage in risk behavior such as walking bare-foot. International travel has increased tremendously in recent years, with >1 billion tourist arrivals worldwide since 2012. As this increase includes developing countries, research into helminth infections among travelers seems justified, especially as asymptomatic infection with helminths can cause morbidity long after the primary infection [8, 11–13]. However, research into prevalence (P), attack rates (AR) and incidence rates (IR) of helminth infections among travelers is scarce. A previous prospective study showed a low risk among short-term travelers (AR: 0.08–0.51%, and IR: 1.1–6.4 per 1000 person-months) [14]. In 2008 though, among 6,957 ill travelers returning to Europe, 156 (2%) were diagnosed with a helminth infection: strongyloidiasis in 54/156 (35%) and loiasis in 10/156 (6%). Schistosomiasis was reported separately, and found in 129/6957 (2%) cases [15]. Data collected within the Geosentinel study among 43,722 ill returning travelers from 1997 to 2004 revealed 271 (0.62%) filarial infections [16]. Africa contributed by far the most to the contracted helminth infections among travelers [16, 17]. Eosinohilia (i.e., >450 eosinophils per μl of blood) is often used as marker pointing to a helminth infection, though reports of eosinophilia coinciding with helminth infections vary considerably [14, 18–22].

Due to the extended period of exposure, one would expect higher attack rates in long-term travelers compared to short-term travelers. In addition, other factors may differ and be of influence on risks, such as compliance with preventive measures and behavioural factors such as as closer contacts with local populations [23–28]. To gain more insight into helminth infections among this specific group of long-term travelers, we focused on four frequently diagnosed helminth infections among ill returning travelers which also have been previously studied among short-term travelers: schistosomiasis, strongyloidiasis, filariasis, and toxocariasis [14–16]. Their primary species, vectors, regions involved, symptoms and numbers infected worldwide are summarized in Table 1. Our aim was to estimate their attack rate and incidence rate among long-term travelers and to investigate factors associated with infection. A second objective was to evaluate the diagnostic relevance of eosinophilia as a predictor for helminth infection.

**Table 1:** Key characteristics of four helminth infections: Schistosomiasis, strongyloidiasis, filariasis, and toxocariasis.

infection		causal agent	reservoir or vector	route of infection	incubation period	general symptoms	regions	estimations of numbers infected/at risk
Schistosomiasis [10, 12, 29]	intestinal	*S*. *mansoni* *S*. *japonicum* *S*. *mekongi* *S*. *guineensis* *S*. *intercalatum*	aquatic snails	Contact with contaminated freshwater sources	2–6 weeks	abdominal pain, diarrhea, blood in stool, hepatomegaly, splenomegaly	Africa, Middle East, Caribbean, parts of South-America, parts of Asia, Corsica (France)	218 million at risk
	urogenital	*S*. *haematobium*				hematuria, fibrosis of bladder/ureter, kidney damage		
Strongyloidiasis [11, 29]		*S*. *stercoralis S*. *fülleborni*	Human, dog, monkey	contact with contaminated soil; direct penetration of human skin by infective larvae	14–30 days	abdominal pain, intermittent/persistent diarrhea, cough, wheezing, chronic bronchitis, pruritis, urticaria	mainly (sub)tropical regions, but also in temperate climates	30–100 million infections
Filariasis [8, 29–33]	lymphatic	*W*. *bancrofti*, *B*. *malayi*, *B*. *timori*	mosquitoes	bites of vector	5–18 months (range 1 month-2 years)	lymphoedema, elephantiasis, hydrocele	mainly regions in Africa and Asia	120 million infections
	onchocerciasis	*O*. *volvulus*	blackflies			severe itching, disfiguring skin conditions, visual impairment including permanent blindness		120 million at risk
	loiasis	*Loa loa*	deerflies			itchy swellings (Calabar swellings), eye worm		> 29 million at risk
	mansonellosis	*M*. *perstans*, *M*. *ozzardi*, *M*. *strepocerca*	midges blackflies			amongst others angioedema, pruritis, fever, headache, neurological symptoms		114–580 million at risk in Africa (*m*.*perstans*)
Toxocariasis [21, 29, 34, 35]		*T*.*canis*	dogs	ingestion of eggs on soil/plants contaminated by dog/cat feces (less frequently by eating undercooked meat containing larvae)	1 week-2 years	systemic, abdominal or respiratory symptoms, sometimes dermatological symptoms as well	worldwide	unknown (estimates for just USA already tens of millions)
		*T*.*cati*	cats					

## Methods

### Study population

This study was conducted as part of a prospective mono-center study of Dutch travelers at the Public Health Service travel clinic in Amsterdam from December 2008 through September 2011. All clients aged ≥ 18 years planning to travel to any subtropical or tropical country for ≥12 and ≤52 weeks were invited to participate. All participants were seen by a medical doctor or nurse who specialized in travel medicine. They were advised according to Dutch National Guidelines on Travelers’ Health Advice, receiving oral and written information about how to avoid mosquito-borne infections [36]. The study protocol was approved by the Medical Ethics Committee of the Academic Medical Center of Amsterdam (MEC 08/064). Participants were included after obtaining their written informed consent.

### Survey methods

At enrollment, a standardized questionnaire in Dutch or English was used to collect pre-departure data on socio-demographics, travel history, vaccination status, and purpose of travel: tourism, work/education, visits to friends and/or relatives (VFR). Participants donated a pre-travel blood sample for serologic testing and were given a digital thermometer (Huikeshoven Medical, Tiel, the Netherlands) and asked to take their temperature if they felt feverish. They were asked to keep a structured, weekly travel diary during travel and two weeks after return, recording their itinerary, signs of disease, physician visits, diagnoses and possible self-treatment. Two to six weeks after return, they completed a short post-travel questionnaire including questions about potential helminth exposure (swimming in a fresh- water source, drinking of unboiled water, walking bare-foot, and having had wounds on feet), and donated a second blood sample for serologic testing.

### Laboratory methods

All blood samples were immediately stored at 6°C. The total leukocyte count and the eosinophil count of both pre-travel and post-travel samples were determined within 24 hours by automated analyzer (Sysmex, Kobe, Japan). Blood samples for serologic testing were centrifuged and frozen at -80°C within 24 hours, to be tested after all participants had returned. Serodiagnosis of *Schistosoma mansoni*, *haematobium*, and *japonicum* was performed using indirect hemagglutination assay (IHA) with adult *S*. *mansoni* worm antigens (Fumouze Laboratories, Levallois-Perret, France) and an enzyme-linked immunosorbent assay (ELISA) with *S*. *mansoni* soluble egg antigens [37]. For *S*. *stercoralis*, an in-house ELISA based on an antigen of *S*. *stercoralis* was used [38]. For filariasis, a commercially available ELISA on microtitration wells sensitized with *Acanthocheilonema viteae* somatic antigens was used (Bordier Affinity Products, Crissier, Switzerland). For toxocariasis, a commercially available ELISA on microtitration wells sensitized with *T*. *canis* E/S larval antigens was used (Bordier Affinity Products, Crissier, Switzerland). Sensitivity and specificity in clinical settings were 100% and 93% for the combined IHA ELISA for schistosomiasis; 93% and 95% for the ELISA for strongyloides; 95% and 98% for the ELISA for filariasis; and 91% and 86% for the ELISA for toxocariasis [37–40].

For participants whose post-travel sample yielded positive test results, corresponding pre-travel samples were also tested. The presence of antibodies in both pre- and post-travel sample was considered suggestive for a previous infection. The presence of antibodies in the post-travel sample together with the absence of antibodies in the pre-travel sample was considered suggestive for an incident infection acquired during travel (i.e., a recent infection). If antibodies were detected in the post-travel samples together with a weak positive test result from the corresponding pre-travel sample, previous infection was assumed.

### Data & statistical analysis

Participants were considered at risk for infection if they were susceptible to the disease (i.e., having a seronegative pre-travel sample) and visited at least one helminth-endemic country. Endemicity was based on information from The Global Infectious diseases and Epidemiology Online Network [29]. Visited countries were analysed at continent-level due to small numbers of visiting participants per country. If a traveler visited more than one continent, the continent in which the traveler spent most time was designated as the visited continent. Attack rates, incidence rates, sensitivity, specificity, positive predictive value, and negative predictive value were calculated as previously described [14]. Eosinophilia was defined as an eosinophil count of ≥ 450 per mm^3^.

The prevalence of previous infection with *Schistosoma* spp., *S*.*stercoralis*, filarial species and/or *Toxocara* spp. and the corresponding 95% confidence intervals were calculated. Logistic regression analysis was used to examine the association between previous infection and the following variables: sex, age, country of birth, and total length of stay at previous travel destinations. Variables with a p-value <0.1 in univariable analysis were included in the multivariable model.

Incidence rates and 95% confidence intervals of infection with *Schistosoma* spp., *S*.*stercoralis*, filarial species, and/or *Toxocara* spp. were calculated. Univariable logistic regression analysis for recent infection was performed using a Generalized Estimating Equation (GEE) model. This model takes account of clustered data, as participants could be at risk for 1 to 4 helminth infections, depending on previous immunity and visited endemic countries. To investigate factors associated with incident infection, we selected the variables sex, age, country of birth, purpose of travel, travel duration, visited continents, number of visited countries, and high-risk behavior. Participants were considered positive for high-risk behavior if they had been swimming in a fresh-water source (schistosomiasis) and/or had been walking bare-foot (strongyloidiasis and toxocariasis). Explorative analysis as to participants’ using anti-helminth medication was performed using the chi-square test for categorical data. Anti-helminth medication against schistosomiasis, strongyloiadiasis, filariasis, and/or toxocariasis include albendazole, mebendazole, praziquantel, ivermectine, and diethylcarbamazine (DEC). A p value <0.05 was considered statistically significant.

Only the time spent in helminth-endemic countries was used as denominator to calculate incidence rates for *Schistosoma* and filarial infection. For those who became infected while traveling, the moment of infection was estimated as the midpoint between their arrival and departure dates in endemic countries. All analyses were conducted using STATA Intercooled version 13 (College Station, TX, USA).

## Results

### Study population

Overall, 685 travelers intended to participate. Of these, 42 (6%) were excluded based on changed travel arrangements, 38 (6%) due to loss to follow-up, and 1 due to a missing post-travel blood sample.

For the remaining 604 participants, the median age was 25 years (interquartile range [IQR]: 23–29), 36% were male, the purpose of travel was predominantly tourism (62%), and the median travel duration was 20 weeks (IQR: 15–25). Seven participants traveled less or more than the intended period of 12–52 weeks. The median interval between return from travel and post-travel blood donation was 25 days (IQR 21–33).

One participant visited Tonga (Oceania) exclusively and was counted as visitor to Asia for simplicity purposes. Of all participants, 494 (82%) traveled to one or more countries endemic for schistosomiasis and 566 (94%) to countries endemic for filariasis.

### Serologic results of previous infection

Thirteen participants (95% CI: 0.68–2.8%) had a previous infection, as they tested both pre- and post-travel-positive for the same helminth infections. In 5 (0.8%) participants, antibodies against *Schistosoma* spp. were found, in 3 (0.5%) antibodies against *S*.*stercoralis*, in 4 (0.7%) antibodies against filarial species, and in 1 (0.2%) antibodies against *Toxocara* spp. (Table 2). Three of the 13 individuals (23%) with a previous helminth infection (one *S*.*stercoralis* infection and two infections with filarial species) did not report being born in a developing country nor previous travel to one. In univariable logistic regression analysis, a previous infection was associated with older age, but in the multivariable model none of factors remained significant.

**Table 2:** Characteristics of 604 long-term travelers attending a Dutch travel health clinic, including prevalence of suggested previous infection with *Schistosoma* spp., *S*.*stercoralis*, filarial species, and/or *Toxocara* spp. from December 2008 to September 2011.

	Total		previous helminth infection		univariable				multivariable			
	no.	%	no.*	P (%)	OR	95% CI		p	OR	95% CI		p
Characteristic						lower	upper			lower	upper	
No. participants	604		13									
Sex												
female	389	64	9 (4,2,2,1)	2	1			0.710				
male	215	36	4 (1,1,2,0)	2	0.80	0.24	2.6					
Median age, y (IQR)	25 (23–29)											
Age, y												
< 24	203	34	4 (1,1,2,0)	2	2.6	0.47	14.4	0.027	2.6	0.47	14.5	0.060
24–29	262	43	2 (1,1,0,0,)	1	1				1			
≥ 30	139	23	7 (3,1,2,1)	5	6.8	1.4	33.6		5.7	1.2	28.8	
Country of birth												
Netherlands	563	93	12 (4,3,4,1)	2	1			0.596				
Other European country/US	26	4	1 (1,0,0,0)	4	1.83	0.23	14.7					
Other	15	2	0 (0,0,0,0)	0	na							
Previous travel destinations												
not Asia	299	50	6 (1,2,3,0)	2	1			0.807				
Asia	305	51	7 (4,1,1,1)	2	1.1	0.38	3.5					
not Africa	379	63	5 (0,1,3,1)	1	1			0.073	1			0.203
Africa	225	37	8 (5,2,1,0)	4	2.8	0.89	8.5		2.1	0.66	6.8	
not Latin America	362	60	6 (3,1,2,0)	2	1			0.311				
Latin America	242	40	7 (2,2,2,1)	3	1.8	0.59	5.3					
Total duration at previous travel destinations												
< 1 months	264	44	5 (0,2,3,0)	2	1			0.791				
1–3 months	116	19	2 (1,0,0,1)	2	0.91	0.17	4.7					
> 3 months	224	37	6 (4,1,1,0)	3	1.4	0.43	4.7					

* In parentheses, number of cases of *Schistosoma* spp., *S*.*stercoralis*, filarial species, and *Toxocara* spp., in that order.

### Serologic results of recent infection

Nine participants acquired 10 recent helminth infections as indicated by a negative pre-travel test and a corresponding positive post-travel test. In 2 participants antibodies against *Schistosoma* spp. were found, in 1 antibodies against *S*.*stercoralis*, in 5 antibodies against filarial species, and in 1 participant antibodies against both filarial and *Schistosoma* spp. were found. Characteristics of the 9 recently infected participants are shown in Table 3. In total, 603 subjects were at risk for toxocariasis; none of them acquired an infection during travel. The attack rate (AR) for *Schistosoma* spp. was 0.61 (3/494) and the incidence rate (IR) was 1.5/1,000 person-months (pm); for *S*.*stercoralis* the AR was 0.17 (6/601) and the IR 0.33/1,000 pm; for filarial species the AR was 1.1 (6/566) and the IR 2.6/1,000 pm (Table 4).

**Table 3:** Characteristics, eosinophil counts, and risk behavior of participants with serologic evidence for infection with *Schistosoma* spp., *S*.*stercoralis*, filarial species, and/or *Toxocara* spp.

serological conversion for*		sex	age in years	country of birth	destinations**	travel duration in weeks	eosinophil count per mm^3^ (proportion of leukocytes)		purpose of travel	number of times swimming in lakes, rivers or streams	drinking of unboiled water from natural sources	walking bare-foot	wounds on feet
							pre-travel	post-travel					
1	strong	M	30	Germany	Argentina, Paraguay, Bolivia, Peru, Colombia, Panama	23	350 (5.3%)	290 (6%)	tourism	>10	yes	no	no
2	schis	F	25	Netherlands	**Uganda**, Tanzania	17	300 (3.3%)	120 (2.9%)	work/education	2–5 (bilharzia +)	yes	don’t know	no
3	schis	M	31	Germany	Indonesia, Malaysia, Thailand, Vietnam, Cambodia	13	60 (1%)	70 (0.8%)	tourism	2–5	no	yes (rarely)	don’t know
4	schis/fil	F	27	Netherlands	Thailand, Malaysia, Singapore, Cambodia, Laos	26	110 (0%)	100 (1.3%)	other	2–5 (Cambodia)	no	yes (rarely)	no
5	fil	F	39	Netherlands	Kenya, **Sudan**	40	60 (0.9%)	220 (4.7%)	work/education	0	no	no	no
6	fil	F	22	Netherlands	**Argentina**, Brazil, Bolivia, Peru	20	150 (3.4%)	190 (1%)	work/education	0	yes	yes (rarely)	no
7	fil	F	24	Netherlands	**Mexico**	43	560 (6.7%)	220	other	1	yes	yes (rarely)	yes
8	fil	M	31	Netherlands	**Congo D.R**.	15	130 (2.9)	210 (3.7%)	work/education	2–5	don’t know	no	no
9	fil	F	28	Netherlands	**Indonesia**	23	150 (2.3%)	100 (1.7%)	work/education	>10	don’t know	no	no

* strong = strongyloidiasis, schis = schistosomiasis, fil = filariasis

** country of primary destination in bold

**Table 4:** Attack and incidence rates of infection with *Schistosoma* spp., *S*.*stercoralis*, filarial species, and/*or Toxocara* spp. among long-term travelers with evidence of seroconversion during travel.

helminth	region	number of seroconversions		susceptibles at risk		person-months of travel		attack rate, % (95% CI, %)			incidence rate per 1000 person-months (95% CI)		
Schistosoma spp.	All regions	3		494		1938		0.61		0.13–1.8	1.5		0.50–4.8
	Asia		2		264		1117		0.76	0.092–2.7		1.8	0.44–7.2
	Africa		1		105		513		0.95	0.024–5.2		1.9	0.27–13.8
	Latin America		0		125		307		0	NA		0	NA
S.stercoralis	All regions	1		601		2958		0.17		0.0042–0.92	0.34		0.048–2.4
	Asia		0		269		1267		0	NA		0	NA
	Africa		0		106		528		0	NA		0	NA
	Latin America		1		226		1163		0.44	0.011–2.4		0.86	0.12–6.1
Filaria spp.	All regions	6		566		2308		1.1		0.39–2.3	2.6		1.16–5.8
	Asia		2		266		1203		0.75	0.091–2.7		1.7	0.42–6.6
	Africa		2		107		520		1.9	0.23–6.6		3.8	0.96–15.4
	Latin America		2		193		585		1.0	0.13–3.7		3.4	0.86–13.7
T. canis	All regions	0		603		2968		0		0–0.61^	0		NA
	Asia		0		271		1274		0	NA		0	NA
	Africa		0		107		533		0	NA		0	NA
	Latin America		0		225		1160		0	NA		0	NA

^ one-sided, 97.5% confidence interval

Overall, 454/601 (76%) participants reported that they had been swimming in fresh water, 228/601 (38%) had been drinking unboiled water from natural sources, and 375/601 (62%) had been walking bare-foot on warm humid soil. If only those participants who had visited schistosomiasis-endemic countries and who reported swimming in fresh water were considered at risk, the AR and IR for schistosomiasis rose slightly (AR 0.81, 95%CI: 0.002–0.024, IR 2.1, 95%CI: 0.43–6.1)

In univariable analysis, an association was found between recent helminth infection and the purpose of travel. Compared to tourists, participants traveling for work/education and VFR/other seroconverted more often for the four studied infections (OR 5.5, 95%CI: 1.1–28.2 and OR 10.5, 95%CI: 1.7–63.3 respectively, p = 0.033).

### Use of anti-helminth medication

Overall, 18 (3%) participants reported having used anti-helminth medication, predominantly albendazole. These 18 participants more often traveled for work/education or to visit friends and relatives than for tourism (5.8% and 3.6% vs 1.6%, p = 0.027). Their travel duration was longer compared to non-users of helminth medication (7.6% (≥26 weeks) vs 0.6% (<16 weeks), 1.9% (16–20 weeks) or 2.7% (21–25 weeks), p = 0.003) and they traveled more often to Africa or Asia than to Latin America (6.5% and 3.3% vs 0.88%, p = 0.016). A doctor was visited by 12 of the 18 participants. Praziquantel was prescribed once post-travel to an asymptomatic traveler whose pre-travel blood sample showed anti-schistosomiasis antibodies. This traveler did however seroconvert for a filariasis (patient number 5, Table 3). The other 8 seroconverters did not report using one of abovementioned anti-helminth medications.

### Eosinophilia

The median pre-travel eosinophil count among the 604 participants was 150 per mm3 (IQR: 90–240), and 28/604 subjects (5%) had a pre-travel eosinophilia. Among the 13 participants with a previous infection, the median pre-travel eosinophil count was 130 per mm3 (IQR: 100–230), and none of them had a pre-travel eosinophilia.

Post-travel, the median eosinophil count was 170 per mm3 (IQR: 100–260), and 38 (6%) had a post-travel eosinophilia. Among the 9 participants with a recent infection, the median post-travel eosinophil count was 190 per mm3 (IQR: 100–220), yet none of these 9 had a post-travel eosinophilia. The sensitivity, specificity, PPV and NPV of eosinophilia in pre-travel samples for previous infection and in post-travel samples for recent infection are described in Table 5.

**Table 5:** Post-travel eosinophilia among long-term travelers during travel, including participants with evidence of infection with *Schistosoma* spp., *S*.*stercoralis* and/or filarial species.

	Total, n	eosinophils median / mm^3^ (IQR)	eosinophil count > 450/mm^3^	≥ 8% eosinophils / total leukocyte count	≥ 10% eosinophils / total leukocyte count
all participants	604	170 (100–260)	38 (6%)	n = 31 (5%)	16 (3%)
recent helminth infection	9	190 (100–220)	n = 0 (0%)	n = 1 (11%)	n = 1 (11%)
sensitivity			0%	11%	11%
specificity			94%	95%	97%
positive predictive value			0%	3%	6%
negative predictive value			98%	99%	99%

## Discussion

In this prospective study of long-term travelers to subtropical and tropical countries, the risk of acquiring one of the studied helminth infections during travel was very low, though quite a lot of travelers used anti-helminth medication. Post-travel eosinophilia was not a good marker for seroconversion during travel.

The found incidence rates and attack rates are in line with two previous prospective studies, although the IRs and ARs in our long-term study are even lower than the previously studied short-term travelers [14, 41]. One of the reasons for the low IRs and ARs is probably the decrease of helminth prevalence due to control and elimination programs in endemic countries, which likely also have led to a lower risk of infection in travelers [2]. In addition, a sizeable number of participants used anti-helminth medication. Although some used the medication against other parasitic infection and not specifically against one of the studied infections, some participants used the medication without reporting any symptoms, suggesting preventive treatment against a suspected helminth infection. Remarkably, the travelers who used anti-helminth medication were predominantly VFRs and study- and work-related travelers, as were the participants in our study who appeared most prone to a helminth infection. Anti-helminth medication might have had a preventive effect, which might have resulted in a slight underestimation of the risk of helminth infection. Considering the possible association of travel purpose and helminth infections, it seems sensible that VFRs and study- and work-related travelers should be specifically informed during pre-travel consultation to avoid high-risk behavior and risks of helminth infections during pre-travel health consultation.

Eosinophilia is frequently associated with helminth infections, and laboratory screening of patients at tropical medicine clinics often include eosinophil counts [16, 19, 21, 22]. The eosinophil count found in symptomatic helminth infections is often very high; in a recent German study they found a median eosinophil count of 981 cells/μl among 71 confirmed infections (range 508–15,100 cells/μl) [22]. However, eosinophilia can arise from other medical conditions, including allergic disorders, and in several studies the diagnostic relevance of eosinophilia as predictor or screening for parasitic infections seemed of limited value [14, 18, 20]. In our study, the PPV of eosinophilia for the four helminth infections under study was very low. We found only 3 travelers with an eosinophil count higher than 981 cells/μl in the pre-travel screening as well as in the post-travel screening, all of these being different participants. Our study should be compared with great caution to retrospective and cross-sectional studies that include eosinophilic screening of symptomatic returning travelers; although it might be valuable in those travelers, our study confirms that screening of western asymptomatic travelers seems of limited value.

Our study has some limitations. First, due to the low number of infections during travel, the sample size is too small to calculate incidences for all four studied helminth infections and for possible risk factors.

Second, some factors could have led to an underestimation of the number of helminth infections during travel. For example, incubation periods can vary widely and could have exceeded the time between travel return and blood donation, leading to an underestimation. However, the median time between return and blood donation was 25 days. We therefore assume that the possible number of infections still in window phase would be negligible. Also, the 13 participants with evidence of a previous infection could have been re-infected with the same helminth during the study period. As the serologic tests we used cannot discriminate between primary and secondary infections, we may have underestimated the number of infections during travel. Furthermore, the used laboratorium kits were not designed for detection of all possible species per helminth. Selection bias may also have occurred, as all participants were seeking pre-travel health advice when recruited. Their health awareness was perhaps higher than average, particularly after receiving oral and written advice about protection against mosquitoes, learning about the study, and agreeing to participate.

Third, cross-reaction with antibodies against other helminth infections cannot be excluded [38, 42], especially as two of previous filarial infections were found among travelers who reported no tropical or subtropical travel abroad before participating in our study. However, this finding could have been an error, as data about previous travels was self-reported. In the absence of a gold standard, additional laboratory diagnostics like stool microscopy could have yielded valuable information, but were not part of the study protocol [18].

Finally, our study did not collect data about nature of travel (adventurous, backpacking, high quality hotels). This could have been of influence on the found incidence rates per continent.

## Conclusions

As far as we know, this is the first prospective study of serology confirmed schistosomiasis, strongyloidiasis, filariasis, and toxocariasis in long-term travelers. We showed that the risk of acquiring one of these infections during travel was very low for long-term travelers. Routine screening of eosinophilia among asymptomatic western travelers appeared to have no value for the four helminths under study.

## Supporting information

S1 Supporting information Travel diary (in Dutch). (PDF)

S2 Supporting information Travel diary (in English). (PDF)

S3 Supporting information Post-travel questionnaire (in Dutch). (PDF)

S4 Supporting information Post-travel questionnaire (in English). (PDF)

S1 Dataset Dataset long-term travelers (de-identified). (XLSX)
