# Supplementary material for: Low incidence of helminth infections (schistosomiasis, strongyloidiasis, filariasis, toxocariasis) among Dutch long-term travelers: A prospective study, 2008-2011
Source: PLoS One. 2018 May 30;13(5):e0197770. doi: 10.1371/journal.pone.0197770 (PMC5976197; doi:10.1371/journal.pone.0197770)
Supplement: S1 Supporting information — (PDF) [file pone.0197770.s001.pdf]

	Week nr.	Week nr.	Week nr.	Week nr.
**Algemeen**				
Datum				
Land(en)				
**Preventieve maatregelen**				
Verblijft u in malariagebied?<br>(volgens GGD, landenkaart)				
Aanbevolen malariatabletten te slikken?				
Heeft u tabletten geslikt volgens schema?<br>Indien ja, welke?				
DEET gebruikt?				
Klamboe gebruikt?				
Gedurende de gehele nacht met airco+afgesloten ruimte geslapen?				
**Klachten**				
Koorts gehad? (hoger dan 38˚C)<br>Hoogst gemeten temperatuur?<br>Hoe? (oraal, onder de oksel of rectaal)				
Hoofdpijn?				
Pijn achter de ogen?				
Spierpijn?<br>(behalve de spierpijn na inspanning)				
Gewrichtspijn?<br>Eén gewricht?<br>Meerdere gewrichten?				
Overgeven?				
Diarree? *Alléén aankruisen* bij minimaal 3x diarree per dag!<br>Indien ja: bloed/slijm?				
Huiduitslag?<br>Zo ja waar?, wat?				
Hoesten langer dan een week?				
Overige klachten<br>Indien ja, welke?				
**Behandeling**				
ORS (Orale rehydratie solutie)				
Antidiarree middel?<br>Welk middel?				
Andere medicatie dan u gewend bent gebruikt?<br>Welk middel?				
Arts bezocht?<br>Indien ja, waarom?				
Diagnose?<br>Behandeling gestart?<br>Indien ja, welke?				
