# Supplementary material for: Low incidence of helminth infections (schistosomiasis, strongyloidiasis, filariasis, toxocariasis) among Dutch long-term travelers: A prospective study, 2008-2011
Source: PLoS One. 2018 May 30;13(5):e0197770. doi: 10.1371/journal.pone.0197770 (PMC5976197; doi:10.1371/journal.pone.0197770)
Supplement: S2 Supporting information — (PDF) [file pone.0197770.s002.pdf]

	Week nr.	Week nr.	Week nr.	Week nr.
**General questions**				
Date				
Country/ Countries on itinerary				
**Preventive measures**				
Risk area for malaria? (see malaria map, LCR)				
Is malaria prophylaxis recommended?				
Did you take malaria prophylaxis as prescribed?<br>Which kind?				
Did you use an insect repellent containing DEET?				
Did you sleep under a bed-net?				
Did you sleep in an air-conditioned room during the entire night, with the windows closed?				
**Symptoms**				
Fever? (above 38ºC)<br>Highest measured temperature?<br>How? (oral, armpit or rectal?)				
Headache?				
Pain behind the eye(s)?				
Muscle ache? (unrelated to physical activity)				
Joint pain? (unrelated to physical activity)<br>One or more joints?				
Did you vomit?				
Diarrhea?<br>Containing blood/ mucus?				
Skin rash?<br>Where?				
Did you cough for more than one week?				
Other symptoms?<br>Which?				
**Treatment**				
Did you use ORS? (Oral Redydration Solution)				
Did you use anti-diarrhoea drugs?<br>Which medication?				
Did you take other medication? (other than your routine medication)<br>Which medication?				
Did you consult a doctor?<br>If so, why?				
What was the diagnosis?<br>Did the doctor start treatment?<br>Which treatment?				
