# Supplementary material for: Low incidence of helminth infections (schistosomiasis, strongyloidiasis, filariasis, toxocariasis) among Dutch long-term travelers: A prospective study, 2008-2011
Source: PLoS One. 2018 May 30;13(5):e0197770. doi: 10.1371/journal.pone.0197770 (PMC5976197; doi:10.1371/journal.pone.0197770)
Supplement: S3 Supporting information — (PDF) [file pone.0197770.s003.pdf]

Vragen na de reis:

- Heeft u in meren, rivieren of stromen gezwommen?
  - ja Zo ja: hoe vaak?(of zoals volg. Vraag): 1 keer
    2-5 keer
    >10 keer
  - nee

  Heeft u ongekookt water uit een natuurlijk bron gedronken?
  - ja
  - nee
  - weet niet

- Heeft u blootsvoets buiten gelopen op warm, vochtige grond?
  - ja (altijd, bijna altijd, helft, zelden, nooit, weet niet)
  - nee
  - weet niet

  Had u wondjes onder de voeten?
  - ja
  - nee
  - weet niet
