# Supplementary material for: Low incidence of helminth infections (schistosomiasis, strongyloidiasis, filariasis, toxocariasis) among Dutch long-term travelers: A prospective study, 2008-2011
Source: PLoS One. 2018 May 30;13(5):e0197770. doi: 10.1371/journal.pone.0197770 (PMC5976197; doi:10.1371/journal.pone.0197770)
Supplement: S4 Supporting information — (PDF) [file pone.0197770.s004.pdf]

Questions after travel:

- Did you swim in lakes, rivers or streams?
  - yes If yes: how often? (or like next question): 1 time
    2-5 times
    >10 times
  - no

  Did your drink unboiled water from natural sources?
  - yes
  - no
  - don't know

- Have you been walking bare-foot outside on warm, humid soil?
  - yes (always, almost always, half of time, rarely, never, don't know)
  - no
  - don't know

Did you have wounds on your feet?
- yes
- no
- don't know
